# Electroencephalogram-Based ConvMixer Architecture for Recognizing Attention Deficit Hyperactivity Disorder in Children

**DOI:** 10.3390/brainsci14050469

**Published:** 2024-05-07

**Authors:** Min Feng, Juncai Xu

**Affiliations:** 1 Nanjing Rehabilitation Medical Center, The Affiliated Brain Hospital, Nanjing Medical University, Nanjing 210029, China; 2 School of Chinese Language and Literature, Nanjing Normal University, Nanjing 210024, China; 3 School of Engineering, Case Western Reserve University, Cleveland, OH 44106, USA

**Keywords:** attention deficit hyperactivity disorder (ADHD), ConvMixer, efficient channel attention (ECA), electroencephalogram (EEG), deep learning, early diagnosis

## Abstract

Attention deficit hyperactivity disorder (ADHD) is a neuro-developmental disorder that affects approximately 5–10% of school-aged children worldwide. Early diagnosis and intervention are essential to improve the quality of life of patients and their families. In this study, we propose ConvMixer-ECA, a novel deep learning architecture that combines ConvMixer with efficient channel attention (ECA) blocks for the accurate diagnosis of ADHD using electroencephalogram (EEG) signals. The model was trained and evaluated using EEG recordings from 60 healthy children and 61 children with ADHD. A series of experiments were conducted to evaluate the performance of the ConvMixer-ECA. The results showed that the ConvMixer-ECA performed well in ADHD recognition with 94.52% accuracy. The incorporation of attentional mechanisms, in particular ECA, improved the performance of ConvMixer; it outperformed other attention-based variants. In addition, ConvMixer-ECA outperformed state-of-the-art deep learning models including EEGNet, CNN, RNN, LSTM, and GRU. t-SNE visualization of the output of this model layer validated the effectiveness of ConvMixer-ECA in capturing the underlying patterns and features that separate ADHD from typically developing individuals through hierarchical feature learning. These outcomes demonstrate the potential of ConvMixer-ECA as a valuable tool to assist clinicians in the early diagnosis and intervention of ADHD in children.

## 1. Introduction

Attention deficit hyperactivity disorder (ADHD) is a neurodevelopmental disorder characterized by persistent inattention, hyperactivity, and impulsivity that adversely affects daily functioning and development [1,2]. ADHD affects approximately 5–10% of school-aged children worldwide [3,4], and early diagnosis and intervention are essential to improve the quality of life of patients and their families [5,6]. However, traditional diagnostic methods such as clinical interviews, behavioral observations, and rating scales can be subjective and time-consuming [7,8,9], highlighting the need for objective and valid diagnostic tools.

Recent advances in machine learning, particularly deep learning, have shown promise in utilizing electroencephalography (EEG) signals to diagnose ADHD [10,11]. EEG is a non-invasive and inexpensive method of measuring electrical activity in the brain, and individuals with ADHD exhibit distinctive patterns compared to typically developing (TD) individuals [12]. Various deep learning architectures including convolutional neural networks (CNNs), recurrent neural networks (RNNs), and long short-term memory (LSTM) networks have been applied to EEG-based ADHD diagnosis with promising results [13,14,15,16,17]. However, these methods often face the challenge of effectively capturing the spatial and channel dependence of EEG signals, which is crucial for accurate diagnosis [18]. Some studies have explored the use of EEG analysis in children with attention-deficit/hyperactivity disorder (ADHD), utilizing various techniques such as spatial normalization, smoothing, and multivariate empirical decomposition [19,20,21]. These studies highlight the lack of biomarkers for ADHD diagnosis and the complexity and challenges associated with capturing the spatial and channel dependence of EEG signals in this disorder. Recent surveys indicate the potential of deep learning techniques in uncovering complex patterns and biomarkers associated with mental disorders from non-invasive brain signals such as EEG [22]. The growing interest in machine learning approaches for precision psychiatry aims to personalize diagnosis and treatment based on individual characteristics and brain activity patterns [23]. Moreover, findings on the use of resting-state EEG functional connectivity to identify symptom dimensions in autism suggest potential parallels to the use of EEG-based deep learning models for characterizing ADHD subtypes or symptom dimensions [24]. These recent advances in EEG and deep learning for mental disorders contextualize the current study within the research landscape and emphasize the importance of developing novel deep learning architectures that can effectively capture the spatial and temporal dependencies in EEG signals for accurate ADHD diagnosis.

ConvMixer, a recently proposed architecture that combines deep and point-by-point convolution [25], has shown great potential in various computer vision tasks due to its ability to efficiently capture spatial and channel information [26,27,28]. Despite its success in computer vision, ConvMixer has not been applied to EEG-based ADHD diagnosis. In addition, attention mechanisms such as efficient channel attention (ECA) have gained significant momentum in deep learning due to their ability to focus on relevant features and suppress irrelevant ones [29,30]. Combining ECA with ConvMixer has the potential to improve the model’s ability to capture discriminative patterns in EEG data for ADHD diagnosis.

To address these research gaps, this study proposes ConvMixer-ECA, a novel deep learning architecture that combines ConvMixer with ECA blocks for the accurate diagnosis of ADHD using EEG signals. The incorporation of the ECA blocks allows the model to adaptively recalibrate the channel features and improves its ability to capture discriminative patterns in EEG data. The main contributions of the work are as follows:

- Proposing ConvMixer-ECA, a novel deep learning architecture that combines ConvMixer with ECA blocks for the accurate EEG-based diagnosis of ADHD.
- Conduct extensive experiments to evaluate the performance of ConvMixer-ECA and demonstrate its superior accuracy compared to state-of-the-art deep learning models.
- Investigate the impact of different attentional mechanisms, in particular ECA, on the performance of ConvMixer and highlight the effectiveness of ECA in improving categorization performance.
- Insights into the feature learning process of ConvMixer-ECA are provided through t-distributed stochastic neighbor embedding (t-SNE) visualization, validating its ability to capture discriminative patterns in EEG data for ADHD diagnosis.

The rest of the paper is organized as follows. Section 2 describes the methodology including the ConvMixer-ECA architecture and its components, with a focus on the integration of ECA blocks. Section 3 describes the experiments and results including the training process, performance evaluation, impact of the attention mechanism, and comparative analysis with other models. Section 4 discusses the results, implications, and limitations of the study. Finally, Section 5 summarizes the paper and outlines future research directions.

## 2. Principles and Methodology

### 2.1. Participants and Data Preprocessing

This study employed an openly accessible EEG dataset obtained from IEEE DataPort. The sample included 121 children aged 7–12 years, comprising 61 children diagnosed with ADHD and 60 children serving as TD controls [31]. The cohort included both male and female participants. The ADHD group consisted of 48 boys and 13 girls with a mean age of 9.62 ± 1.75 years, while the TD control group included 50 boys and 10 girls with a mean age of 9.85 ± 1.77 years. The ADHD diagnoses were confirmed by a qualified psychiatrist in accordance with the criteria outlined in the *Diagnostic and Statistical Manual of Mental Disorders* (4th ed., DSM-IV) [32]. The participants in the ADHD group had been undergoing treatment with Ritalin for a period not exceeding 6 months prior to their involvement in the study. On the other hand, the control group consisted of individuals without psychiatric disorders, epilepsy, or any reported involvement in high-risk behaviors. This group was drawn from two sources: 50 boys were selected from a primary school, while 10 girls were chosen from an all-girls’ primary school. Following assessment by a child and adolescent psychiatrist, it was confirmed that none of the children in the control group displayed any psychiatric issues.

**Figure 1:**
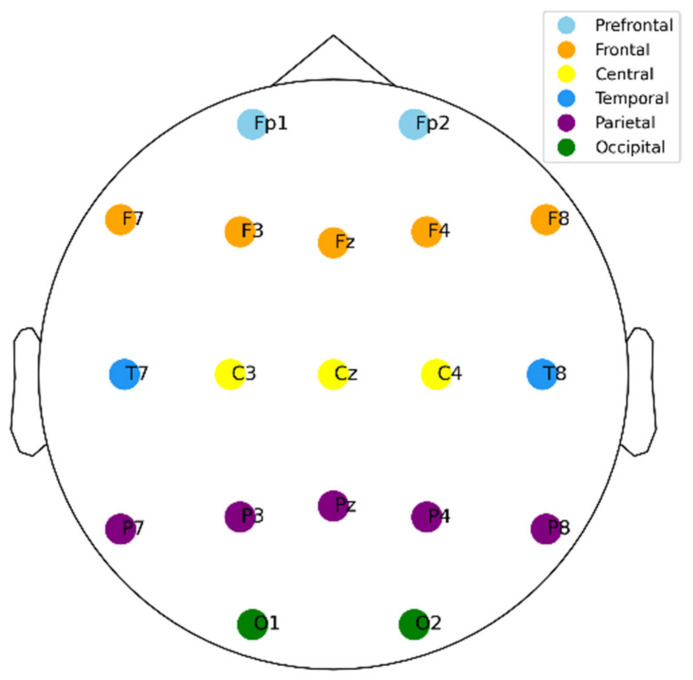
The 19-channel EEG recording based on the International 10–20 system.

**Figure 2:**
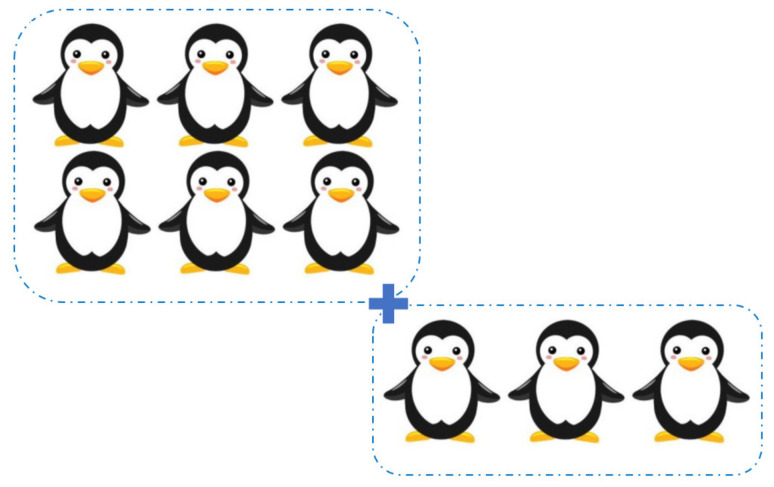
An example of the pictures displayed to the children.

EEG recordings were performed with the International 10–20 system, consisting of 19 channels: Fz, Cz, Pz, C3, T3, C4, T4, Fp1, Fp2, F3, F4, F7, F8, P3, P4, T5, T6, O1, and O2 (Figure 1) [33]. Recordings were sampled at a frequency of 128 Hz and reference electrodes A1 and A2 were placed on the participants’ earlobes. The EEG recording protocol consisted of tasks designed to elicit visual attention, thus assessing the main deficits in children with ADHD. During the task, participants were presented with images of cartoon characters and asked to count them (Figure 2). The number of characters presented ranged from 5 to 16, and the image size was calibrated to ensure legibility. Images were presented sequentially, and each subsequent image was displayed immediately after the participant responded, with the duration of the EEG recording session depending on the participant’s performance and response rate.

Preprocessing is critical in EEG signal processing to ensure data quality and reliability for subsequent analysis by removing artifacts, reducing noise, and separating relevant frequency bands. This study focused on two key preprocessing techniques: the Butterworth bandpass filter and the segmentation process. EEG signals were subjected to preprocessing using a sixth-order Butterworth bandpass filter with a frequency range of 4–45 Hz. This was conducted in order to remove low-frequency drift and high-frequency noise while preserving the relevant EEG frequency bands. The filter coefficients were determined based on the filter order and cutoff frequencies. The lower and upper cutoff frequencies were set at 4 Hz and 45 Hz, respectively. Following filtration, the continuous EEG signals were divided into 4-s epochs, with each epoch representing a specific temporal window of the EEG recording.

The Butterworth bandpass filter is a widely used technique in signal processing to extract a specific range of frequencies from a signal. In EEG analysis, it removes unwanted frequencies and artifacts that may interfere with the desired brain activity. The transfer function of a sixth-order Butterworth bandpass filter is given by:

(1) $$H(s)=\frac{\omega_{c1}\cdot \omega_{c2}}{s^{6}+a_{1}\cdot s^{5}+a_{2}\cdot s^{4}+a_{3}\cdot s^{3}+a_{4}\cdot s^{2}+a_{5}\cdot s+a_{6}}$$

where $\omega_{c1}$ and $\omega_{c2}$ are the lower and upper cutoff frequencies, and $a_{1}$, $a_{2}$, $a_{3}$, $a_{4}$, $a_{5}$, and $a_{6}$ are the filter coefficients determined by the filter order and cutoff frequencies. In this study, a Butterworth bandpass filter was designed with a frequency range of 4–45 Hz. This range was selected to remove low-frequency drift and high-frequency noise while preserving the relevant EEG frequency bands. The rationale for this approach was to capture the primary activity associated with the P300 component and other cognitive processes of interest in the study of ADHD, while minimizing the influence of artifacts [34,35].

The segmentation process divides a continuous EEG signal into manageable segments or epochs; this process is essential for further analysis and feature extraction. The segmentation process can be mathematically represented as:

(2) $$x_{i}(n)=x(n+(i-1)\cdot N),\;\mathrm{for}\;n=1,2,\ldots ,N$$

where $x_{i}(n)$ represents the i-th segment of the EEG signal, x(n) is the original EEG signal, and N is the number of samples in each segment, determined by the sampling frequency and segment duration.

In this study, the dataset was divided into a training set and a testing set, each of which contained EEG epochs from two distinct groups of subjects. Notably, the training and testing epochs were extracted from different subjects to ensure that there was no overlap between the sets. Each EEG signal was segmented into 4-s epochs. The preprocessing steps cleaned, filtered, and prepared the EEG dataset for further analysis. The Butterworth bandpass filter effectively removed unwanted frequencies and artifacts, and the segmentation process divided the continuous EEG signal into manageable epochs, ready for feature extraction and classification tasks.

### 2.2. ConvMixer Architecture

The ConvMixer architecture is a groundbreaking approach to visual modeling that seamlessly integrates the strengths of CNNs and transformers. This innovative architecture employs a series of strategic design choices to effectively process and learn from visual data. The key strategies adopted by ConvMixer include patch embedding, a ConvMixer layer, a mixing mechanism, layer stacking, and global pooling [25].

Patch embedding: The input image is divided into fixed-size patches, which are then mapped to a high-dimensional space. This process is mathematically represented as:

(3) $$N=\frac{HW}{P^{2}}$$

where H, W, and P denote the height, width, and patch size of the image, respectively. Each patch is flattened and linearly projected using an embedding matrix $W_{e}$ to obtain the embedded patch matrix E:

(4) $$E=\mathrm{Reshape}(X)\cdot W_{e}$$

where $X\in {\mathbb{R}}^{H\times W\times C}$ represents the input image, Reshape(⋅) represents the operation of dividing the image into patches and flattening them, and $W_{e}\in {\mathbb{R}}^{(P^{2}C)\times D}$ represents the embedding matrix.

ConvMixer layer: The core building block of ConvMixer is a ConvMixer layer, which consists of depthwise separable convolutions and pointwise convolutions. Depthwise convolutions capture local spatial relationships within each channel of the input tensor X:

(5) $$X_{d}=\mathrm{DepthwiseConv}(X,W_{d})$$

where X represents the input tensor, $W_{d}$ represents the depthwise convolution kernel, and $X_{d}$ represents the output of the depthwise convolution. Pointwise convolutions, applied subsequently, mix information across channels:

(6) $$X_{p}=\mathrm{PointwiseConv}(X_{d},W_{p})$$

where $W_{p}$ represents the pointwise convolution kernel, and $X_{p}$ represents the output of the pointwise convolution.

Mixing mechanism: ConvMixer employs a mixing mechanism that applies depthwise separable convolutions and pointwise convolutions independently to each patch:

(7) $$p_{i}^{d}=\mathrm{DepthwiseConv}(p_{i},W_{d})$$

(8) $$p_{i}^{p}=\mathrm{PointwiseConv}(p_{i}^{d},W_{p})$$

where $p_{i}$ represents the i-th patch, $p_{i}^{d}$ denotes the output of the depthwise convolution, and $p_{i}^{p}$ represents the output of the pointwise convolution. The mixed output is represented as:

(9) $$P=[p_{1}^{p};p_{2}^{p};\cdots ;p_{N}^{p}]$$

where $P\in {\mathbb{R}}^{N\times D}$ represents the matrix of mixed patches.

Layer stacking: To learn hierarchical feature representations, ConvMixer stacks multiple ConvMixer layers together:

(10) $$X^{\left(l+1\right)}=\mathrm{ConvMixerLayer}(X^{\left(l\right)})$$

where $X^{\left(l\right)}$ represents the output of the l-th ConvMixer layer, and $X^{\left(l+1\right)}$ represents the output of the (l+1)-th ConvMixer layer.

Global pooling and classification: After processing through multiple ConvMixer layers, the model performs global average pooling (GAP) on the features of all patches to obtain a pooled feature vector:

(11) $$f=\frac{1}{N}\sum_{i=1}^{N}p_{i}^{p}$$

where $p_{i}^{p}$ represents the pointwise convolution output of the i-th patch, and $f\in {\mathbb{R}}^{D}$ represents the pooled feature vector. Finally, the pooled features are fed into a classifier for prediction.

### 2.3. ECA Mechanism

The ECA mechanism is a groundbreaking approach in deep learning that refines channel-wise attention within CNNs while maintaining a balance between model complexity and performance [29]. ECA employs several key strategies.

GAP: GAP is applied to the input feature map $X\in {\mathbb{R}}^{C\times H\times W}$ to obtain a vector $z\in {\mathbb{R}}^{C}$ that encapsulates channel-wise information:

(12) $$z_{c}=\frac{1}{H\times W}\sum_{i=1}^{H}\sum_{j=1}^{W}x_{c,i,j}$$

Adaptive kernel size determination: ECA determines the kernel size for the one-dimensional convolution based on the number of channels C:

(13) $$k=\psi (\frac{C}{\gamma })+1$$

where ψ(⋅) is the rounding operation and γ is a hyperparameter.

Convolution: A convolution with kernel size k is applied to z to generate attention weights $\hat{z}\in {\mathbb{R}}^{C}$:

(14) $${\hat{z}}_{c}=\sum_{i=1}^{k}w_{i}\cdot z_{\mathrm{mod}(c+i-\left\lfloor \frac{k}{2}\right\rfloor -1,C)+1}$$

where $w_{i}$ are learnable convolution weights.

Sigmoid activation: A sigmoid activation function is applied to $\hat{z}$ to obtain the final attention weights $a\in {\mathbb{R}}^{C}$:

(15) $$a_{c}=\frac{1}{1+e^{-{\hat{z}}_{c}}}$$

Channel-wise attention scaling: The input feature map X is scaled with the attention weights a to obtain a refined feature map $\tilde{X}\in {\mathbb{R}}^{C\times H\times W}$:

(16) $${\tilde{X}}_{c,i,j}=a_{c}\cdot X_{c,i,j}$$

ECA enhances the representational capacity of CNNs while maintaining efficiency. The mathematical formulations provide a clear understanding of the operations involved, facilitating integration into various CNN architectures for improved feature learning and task-specific adaptability.

### 2.4. Implementation of ConvMixer-ECA

ConvMixer-ECA combines the ConvMixer model with the ECA mechanism to enhance the model’s ability to capture and refine channel-wise attention. The ConvMixer-ECA architecture consists of patch embedding, a stack of ConvMixer blocks with integrated ECA blocks, GAP, layer normalization, and a fully connected layer for classification.

Patch embedding: The input tensor $X\in {\mathbb{R}}^{B\times C\times L}$ is divided into patches and projected into a higher-dimensional space using a convolution operation:

(17) $$X_{p}=\mathrm{Conv}(X,W_{p},p)$$

where $X_{p}\in {\mathbb{R}}^{B\times C_{p}\times L_{p}}$ is the patch embedding, $W_{p}\in {\mathbb{R}}^{C_{p}\times C\times p}$ is the patch embedding kernel with size p, and Conv(⋅) denotes the convolution operation.

ConvMixer block with ECA: Each ConvMixer block consists of a depthwise convolution, Gaussian error limit unit (GELU) activation, pointwise convolution, and an ECA block for channel-wise attention refinement:

(18) $$X^{\prime }=X+\mathrm{ECABlock}(\mathrm{Conv}(\mathrm{GELU}(\mathrm{DepthwiseConv}(X,W_{d})),W_{p}))$$

where $X^{\prime }\in {\mathbb{R}}^{B\times C_{p}\times L_{p}}$ is the output of the ConvMixer block, $W_{d}\in {\mathbb{R}}^{C_{p}\times 1\times k}$ is the depthwise convolution kernel with size k, $W_{p}\in {\mathbb{R}}^{C_{p}\times C_{p}\times 1}$ is the pointwise convolution kernel, and ECABlock(⋅) represents the ECA block.

The ECA block applies GAP, convolution, and sigmoid activation to generate channel-wise attention weights:

(19) $$z=\frac{1}{L}\sum_{i=1}^{L}X_{i}$$

(20) $$\hat{z}=\mathrm{Conv}(z,W,k)$$

(21) $$a=\sigma (\hat{z})$$

where $z\in {\mathbb{R}}^{B\times C\times 1}$ is the channel-wise descriptor, $\hat{z}\in {\mathbb{R}}^{B\times C\times 1}$ is the output of the convolution, $a\in {\mathbb{R}}^{B\times C\times 1}$ represents the channel-wise attention weights, and σ(⋅) is the sigmoid activation function.

GAP and classification: The output of the ConvMixer blocks is subjected to GAP to obtain a global representation:

(22) $$y=\frac{1}{L_{p}}\sum_{i=1}^{L_{p}}{X^{\prime }}_{i}$$

where y is the global representation.

The global representation is then normalized using layer normalization and passed through a fully connected layer for classification:

(23) $$\dot{y}=\mathrm{LayerNorm}(y)$$

(24) $$\hat{y}=\mathrm{FC}(\dot{y},W_{fc})$$

where $\hat{y}\in {\mathbb{R}}^{B\times N}$ is the output of the fully connected layer, $W_{fc}\in {\mathbb{R}}^{C_{p}\times N}$ is the fully connected weight matrix, and N is the number of classes.

ConvMixer-ECA uses deep convolution, point-by-point convolution, and ECA blocks to capture local and global dependencies while efficiently focusing on correlated channels. Mathematical formulas clearly illustrate the operations and transformations used to process and optimize the input data to produce accurate predictions.

**Figure 3:**
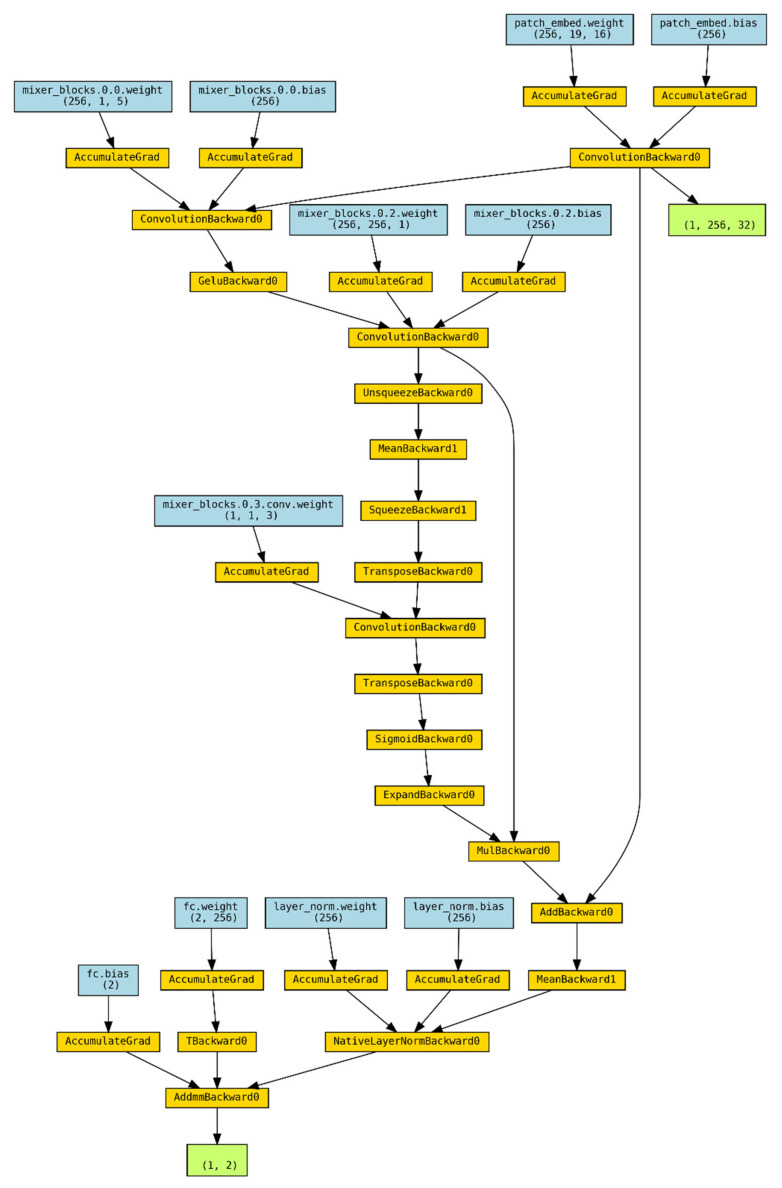
The ConvMixer-ECA architecture.

As shown in Figure 3, ConvMixer-ECA extends the ConvMixer architecture by combining ECA blocks. The model consists of a patch embedding layer, followed by a series of mixer blocks, and finally a classification layer. While ConvMixer-ECA is typically implemented at depth 8, Figure 3 shows the model at depth 1 for clarity.

The patch embedding layer is realized by a one-dimensional (1D) convolutional layer that transforms the input tensor into patches and projects them into a higher dimensional space. The heart of the model lies in the mixer blocks, each of which consists of three key components: a deep convolutional layer for spatial mixing, a point-by-point convolutional layer for channel mixing, and an ECA block for channel feature recalibration. The ECA block uses adaptive mean pooling and a 1D convolutional layer to capture cross-channel interactions and generate attention weights that are multiplied by the input features to emphasize the most relevant channels.

After the mixing block, the feature map is subjected to global average pooling (GAP) and layer normalization to generate a compact and normalized representation. Finally, a fully connected layer generates output logits for classification.

ConvMixer-ECA is initialized with several key hyperparameters including the number of input channels, number of categories, patch size, hidden dimension, depth, and kernel size. During the forward pass, the inputs are processed sequentially through the patch embedding layer, the mixer block, the GAP, the layer normalization, and the fully connected layer.

In essence, ConvMixer-ECA seamlessly combines the strengths of the ConvMixer architecture with the capabilities of the ECA block. This synergistic combination enables the model to efficiently process input data through deep and point-by-point convolution while enhancing its ability to capture and exploit channel dependencies. The final architecture strikes a balance between computational efficiency and representational power, making it well-suited for a wide range of computer vision tasks.

## 3. Experiments and Results

### 3.1. Training ConvMixer-ECA

To ensure that there was no overlap between the training and test sets, data from different subjects were assigned to each set. The training set came from 80% of the subjects and consisted of 3352 samples, while the test set came from the remaining 20% of the subjects and consisted of 821 samples. A total of 70% of the training data was used to train ConvMixer-ECA, and the remaining 30% of the data was used to validate the training process. Although the proportions of the training and validation protocols were the same, different data segments were selected for each training cycle due to the random interruption of the data.

Binary cross entropy (BCE) was used as the loss function for training, and the Adam optimizer was used to determine the weights and filters for the network, minimizing the loss function using a back-propagation technique with a learning rate of 0.0001. The total number of cycles was set to 30, and the number of iterations per cycle depended on the batch size of 64, as described above.

To evaluate the training performance on the training and validation sets, the loss and accuracy of the validation set were calculated at the end of each cycle and in the final iteration. All implementations were performed on an *NVIDIA V100 GPU* (NVIDIA Corporation, Santa Clara, CA, USA) with *32 GB of DDR4 RAM* (Samsung Group, Seoul, Republic of Korea) running *Ubuntu 20.04.1 LTS* (Canonical Ltd., London, UK) and *Python 3.11.6* (Python Software Foundation, Wilmington, NC, USA).

**Figure 4:**
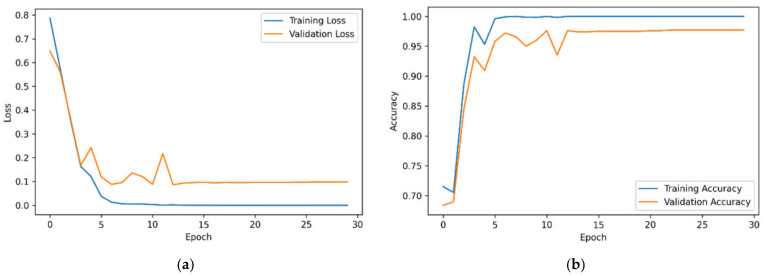
Training and validation of the ConvMixer-ECA model: (**a**) loss and (**b**) accuracy across the epochs.

The training results show that the model exhibited good performance and convergence over 30 cycles (Figure 4). The steady decrease in training loss from about 0.7 to 0.1, along with a corresponding decrease in validation loss, indicates that the model learns effectively from the training data and generalizes well to unseen data. The continued improvement in training and validation accuracy to 0.98 and 0.95, respectively, further highlights the model’s success in learning to correctly categorize samples. Overall, the results show a well-trained model that performed well on both the training and validation sets, demonstrating its effectiveness in learning from the given data and generalizing to unseen samples.

### 3.2. Results and Analysis

The performance of ConvMixer-ECA was evaluated with several metrics including the accuracy, the F1 score, recall, and precision [36]. These metrics are defined by the following equations:

(25) $$Accuracy=\frac{TP+TN}{TP+TN+FP+FN}$$

(26) $$Precision=\frac{TP}{TP+FP}$$

(27) $$Recall=\frac{TP}{TP+FN}$$

(28) $$F1=\frac{2\times TP}{2\times TP+FP+FN}$$

where TP is the number of instances correctly predicted as positive, TN is the number of instances correctly predicted as negative, FP is the number of instances incorrectly predicted as positive, and FN is the number of instances incorrectly predicted as negative.

The performance of the trained ConvMixer-ECA model was evaluated on a test set consisting of 821 samples from 20% of the subjects not used for training. The model achieved 94.52% accuracy, 94.69% precision, 96.40% recall, and 95.54% F1 score. These metrics indicate strong performance, with the model correctly identifying the majority of positive instances while maintaining a low false positive rate.

**Figure 5:**
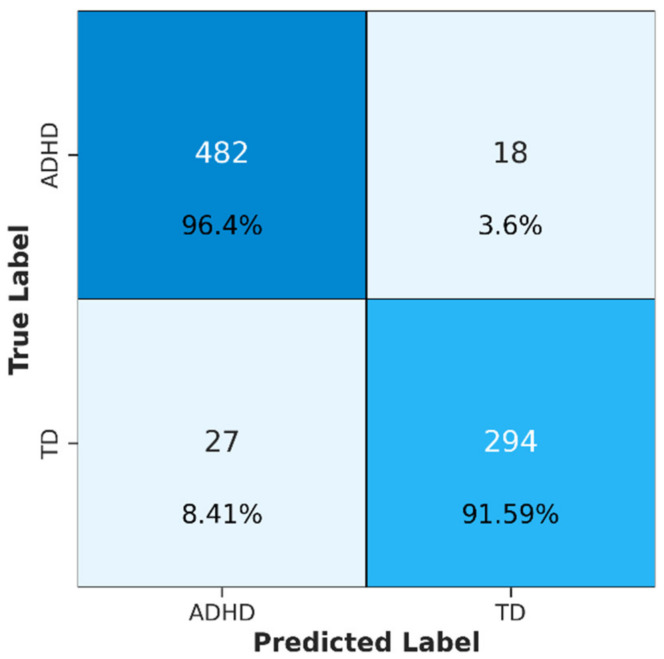
The confusion matrix for ADHD and TD classification.

The confusion matrix shown in Figure 5 provides a detailed assessment of the performance of the trained ConvMixer-ECA model in classifying ADHD and TD individuals. The model demonstrates strong classification ability with a true positive rate of 96.4% for the ADHD cases and a true negative rate of 91.59% for the TD cases. The low false negative rate of 3.6% indicates the high sensitivity of the model in detecting ADHD cases. The ConvMixer-ECA architecture demonstrated its effectiveness in capturing the distinguishing features of ADHD as evidenced by the high accuracy in correctly identifying ADHD cases. The model’s ability to discriminate between ADHD and non-ADHD cases is further supported by its high true negative rate.

To enhance the robustness of the performance evaluation, we implemented a 5-fold cross-validation procedure in testing the ConvMixer-ECA model. The dataset was randomly partitioned into five equally sized folds, ensuring a balanced representation of both ADHD and TD subjects. In each iteration, four folds were used for training, and the remaining fold was used for testing, with the process repeated five times. The accuracy results were obtained from the 5-fold cross-validation (Table 1), yielding an average accuracy of 0.9290. The consistent accuracy values across the different folds demonstrated the stability and robustness of the model’s performance, validating the effectiveness of the ConvMixer-ECA architecture in accurately classifying ADHD and TD subjects based on their EEG data. By employing this rigorous methodological approach, we enhanced the reliability of the reported results and strengthened the evaluation of the model’s performance.

**Figure 6:**
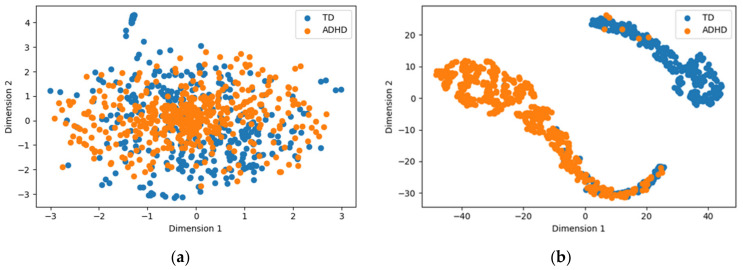
Visualizing the t-SNE-reduced outputs of the proposed model’s (**a**) first layer and (**b**) final layer.

To further analyze the functionality of ConvMixer-ECA, t-SNE visualizations of the model’s first and last layer outputs were generated, revealing the separation of ADHD within the learned feature space. Significant improvements were seen in the ADHD and TD samples (Figure 6). The first layer showed initial discriminative ability, although there was some overlap between categories. In contrast, the last layer showed significant separation, highlighting the hierarchical feature learning process of the model. The clear separation observed in the last layer validates the ability of ConvMixer-ECA in ADHD diagnosis, successfully capturing the underlying patterns that distinguish ADHD individuals from TD individuals. In summary, the t-SNE output demonstrates the model’s ability to learn increasingly discriminative features, and the final layer confirms its effectiveness in distinguishing between the two categories.

**Figure 7:**
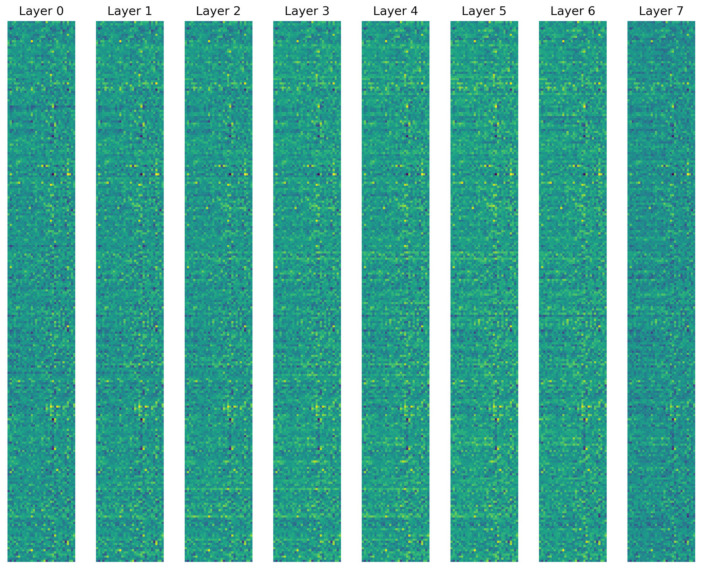
Activation maps from each ConvMixer block layer.

The activation maps of all layers of the ConvMixer-ECA model, as visualized in Figure 7, demonstrate the progressive feature extraction process. The initial layer (Layer 0) captures local patterns and textures after the patch embedding. As ConvMixer blocks progress from Layer 1 to Layer 7, the model learns and refines features through the use of depthwise convolutions to capture the spatial relationships within channels, pointwise convolutions to mix information across channels, and ECA blocks to adaptively recalibrate channel-wise attention. The activation maps become increasingly abstract and capture higher-level features in the deeper layers, as evidenced by the transition from fine-grained, dense activations in the earlier layers to coarse-grained, sparse activations in the deeper layers. This suggests that the deeper layers are selective to specific higher-level features and capture more complex and semantically meaningful patterns. The final ConvMixer block (Layer 7) outputs a rich set of discriminative features that are then aggregated and used for classification. This hierarchical feature learning process, where earlier layers learn low-level features and deeper layers progressively learn more abstract representations, enables the ConvMixer-ECA model to effectively identify the distinguishing characteristics of ADHD and TD samples, leading to accurate classification. These findings indicate that the model is capable of effectively capturing the underlying patterns and features that distinguish individuals with ADHD from those without, thereby achieving accurate categorization.

### 3.3. The Impact of Attention Mechanisms on ConvMixer Performance

A comprehensive analysis was performed to investigate the impact of different attention mechanisms on the performance of ConvMixer. The attention mechanisms evaluated include the convolutional block attention (CBA) module, squeeze and excite attention (SEA), non-localized attention (NLA), and ECA. Each ConvMixer variant with a specific attention mechanism is trained and evaluated on the same dataset, with the original ConvMixer model serving as the benchmark for comparison.

Table 2 shows the evaluation metrics (i.e., accuracy, precision, recall, and F1 score) for each ConvMixer variant. The baseline ConvMixer model achieved an accuracy of 0.9160, providing a reference point for assessing the impact of attentional mechanisms. The ConvMixer-CBA with CBA improved slightly over the baseline, achieving an accuracy of 0.9196. The ConvMixer-SEA further improved the performance to an accuracy of 0.9318. In addition, ConvMixer-NLA showed excellent results with an accuracy of 0.9415. However, ConvMixer-ECA achieved the highest performance among the variants with an accuracy of 0.9452.

The experimental results provide compelling evidence that the combination of attentional mechanisms consistently improved ConvMixer’s performance across all evaluation metrics. In particular, ConvMixer-ECA has emerged as the most effective method, highlighting the potential of ECA to significantly improve the model’s classification or prediction capabilities. These results underscore the importance of exploring and exploiting attentional mechanisms to improve the performance of deep learning models. The integration of attentional mechanisms, particularly ECA, shows promise for improving the effectiveness of ConvMixer.

### 3.4. Comparative Evaluation of Recognition Models

ConvMixer-ECA was compared with five commonly used deep learning models (EEGNet, CNN, RNN, LSTM, and gated recurrent unit (GRU)) to evaluate its effectiveness in EEG signal classification. EEGNet, a compact CNN architecture specifically designed for EEG-based brain–computer interfaces [37], has shown promising results in a variety of EEG classification tasks.

**Figure 8:**
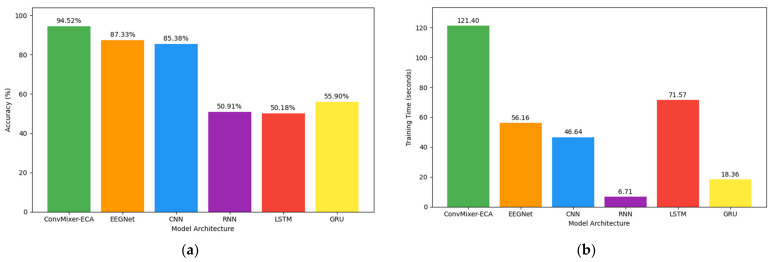
Comparison of the model architectures: (**a**) accuracy and (**b**) training time.

As shown in Figure 8a, ConvMixer-ECA achieved the highest accuracy of 94.52%, which was 7.19 percentage points higher than the second best model, EEGNet. CNN, RNN, and LSTM achieved 85.38%, 50.91%, and 50.18% accuracy, respectively, while GRU had the lowest accuracy of 55.90%.

The excellent performance of ConvMixer-ECA can be attributed to its combination of deep convolution, pooling, and ECA mechanisms, which improves its discriminative power and ability to learn salient features from EEG data. In contrast, recurrent models (RNN, LSTM, and GRU) performed poorly, possibly due to the difficulty in capturing long-term dependencies in EEG signal sequences. This comparative analysis establishes ConvMixer-ECA as a promising architecture for accurate EEG-based recognition, outperforming specialized EEG classification models such as EEGNet and several standard deep learning architectures.

A comparative analysis of computational time for different deep learning models was conducted on a platform comprising an Intel Core i5-9500T (Intel Corporation, Santa Clara, CA, USA) processor with 16 GB of DDR4 RAM running Ubuntu 20.04.1 LTS and Python 3.11.6. The training condition was set to 10 epochs.

As illustrated in Figure 8b, the outcomes indicate that ConvMixer-ECA and LSTM were more computationally demanding, whereas GRU and RNN were the most efficient in terms of training time on the specified platform and configuration. Although ConvMixer-ECA necessitated more computational resources in comparison to the other models, the hardware configuration utilized in this analysis was sufficient to accommodate its training requirements, rendering it a viable option for use in a PC environment.

## 4. Discussion

The experimental results of this study demonstrate the effectiveness of the proposed ConvMixer-ECA architecture in detecting ADHD using EEG signals. The model achieved a remarkable accuracy of 94.52%, outperforming several state-of-the-art deep learning models including EEGNet, CNN, RNN, LSTM, and GRU. The high precision (94.69%), recall (96.40%), and F1 score (95.54%) further emphasize the model’s ability to correctly identify ADHD cases while maintaining a low false-positive rate ability.

The t-SNE visualization of the model layer output provides valuable insight into the feature learning process, demonstrating the model’s ability to learn increasingly discriminative features. This hierarchical feature learning process demonstrates the effectiveness of ConvMixer-ECA in capturing the underlying patterns and features that distinguish ADHD from TD.

The effects of different attentional mechanisms on the performance of the ConvMixer model were extensively investigated. The combination of attentional mechanisms consistently improved the model’s performance, and ConvMixer-ECA using ECA achieved the highest accuracy among the variants. This finding highlights the importance of exploring and exploiting attentional mechanisms to improve the classification capabilities of deep learning models in EEG-based recognition tasks.

The proposed ConvMixer-ECA method, summarized in Table 3, showed excellent performance in identifying ADHD using EEG signals compared to the existing literature. The excellent performance of ConvMixer-ECA can be attributed to its ability to efficiently capture spatial and channel dependencies in EEG signals through a combination of deep convolution, pooling, and ECA mechanisms.

The results of this study have important implications for the early diagnosis and intervention of ADHD in children. The high accuracy and reliability of ConvMixer-ECA demonstrates its potential as a valuable tool to assist clinicians in the diagnostic process, facilitating timely intervention and individualized treatment plans. The ConvMixer-ECA model can capture EEG abnormalities associated with ADHD such as increased theta activity, reduced beta activity, and altered fronto-central connectivity [41,42]. These abnormalities are robust markers of ADHD and are linked to impaired inhibitory control and hypoarousal in the prefrontal cortex [43]. Functional imaging studies have also highlighted abnormal functioning in the prefrontal cortex and striatum in individuals with ADHD [44]. Moreover, there is evidence of altered fronto-subcortical intrinsic functional connectivity in ADHD. The model’s ability to extract complex patterns and relationships from EEG data may potentially reveal the distinct EEG signatures of ADHD, providing valuable insights for diagnostic and therapeutic purposes.

**Figure 9:**
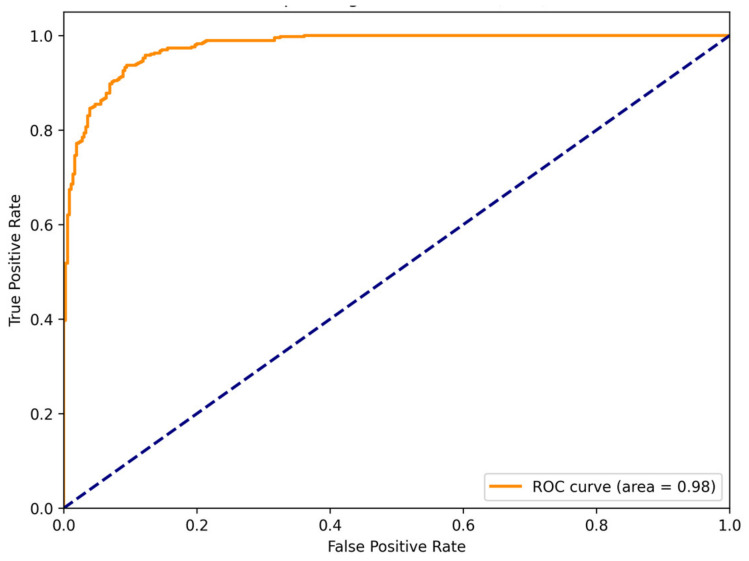
Receiver operating characteristic (ROC) curve of the ConvMixer-ECA model.

The present study has some limitations that need to be addressed in future studies. The false positive rate of 8.41% suggests that there is room for improvement in reducing the misclassification of TD individuals as ADHD individuals. A comparison of the performance of our EEG-based ConvMixer-ECA model with that of non-EEG-based ADHD diagnostic tools such as clinical interviews, behavioral assessments, and neuropsychological tests could provide a more comprehensive understanding of its potential utility and complementary role in the diagnostic process. However, the ROC curve analysis, with an area under the curve (AUC) of 0.98, demonstrated the high overall discriminative ability of our ConvMixer-ECA model in distinguishing between ADHD and TD children (Figure 9). This strong performance highlights the potential of our approach to assist clinicians in the accurate diagnosis of ADHD, while future work should focus on further improving the model’s specificity to minimize false positives. Subsequent research endeavors should also explore the specificity and applicability of the model to various EEG classification tasks such as distinguishing between ADHD subtypes or predicting treatment response. Investigating the generalizability of the ConvMixer-ECA model by validating its performance on larger and more diverse ADHD datasets including subjects from different age groups, ethnicities, and clinical subtypes would further establish its robustness. Furthermore, the integration of multimodal data sources such as functional magnetic resonance imaging (fMRI) or genetic information with EEG signals could facilitate the development of more comprehensive and accurate models for the diagnosis and subtyping of ADHD. Moreover, future research should focus on further validating and refining the model for broader clinical utility, facilitating its integration into clinical practice and decision-making processes.

## 5. Conclusions

This study proposes ConvMixer-ECA, an innovative deep learning framework for identifying ADHD from EEG signals. The architecture integrates the ConvMixer model, which uses deep convolution and point-by-point convolution for optimized feature extraction, combined with ECA blocks to recalibrate features in a channelized manner. The model was trained and evaluated on EEG data from 60 healthy children and 61 children with ADHD. Experiments and analyses led to the following main conclusions:

(1) ConvMixer-ECA performed well in detecting ADHD with an accuracy of 94.52%. This highlights its effectiveness in recognizing discriminative features and accurately classifying ADHD individuals from TD individuals.
(2) The integration of attentional mechanisms, especially ECA, significantly improved the performance of the ConvMixer model. It outperformed other attention-based variants, highlighting the importance of incorporating attentional mechanisms in EEG-based recognition tasks.
(3) ConvMixer-ECA outperformed existing state-of-the-art deep learning models including EEGNet, CNN, RNN, LSTM, and GRU, establishing ConvMixer-ECA as an accurate EEG-based ADHD detection method.
(4) The t-SNE visualization of the output of the ConvMixer-ECA layer confirmed the model’s ability to learn to distinguish between the intrinsic patterns and features of individuals with ADHD and those with TD through hierarchical feature learning.

These results highlight the potential of ConvMixer-ECA as a valuable tool to assist clinicians in the early diagnosis and intervention of ADHD in children.

## Figures and Tables

**Table 1:** Accuracy from the 5-fold cross-validation of the ConvMixer-ECA.

Fold	Fold 1	Fold 2	Fold 3	Fold 4	Fold 5
Accuracy	0.9245	0.9038	0.9567	0.9245	0.9354

**Table 2:** Evaluation results of ConvMixer variants with different attention mechanisms.

Model	Accuracy	Precision	Recall	F1 Score
ConvMixer	0.9160	0.9016	0.9544	0.9273
ConvMixer-CBA	0.9196	0.9022	0.9610	0.9307
ConvMixer-SEA	0.9318	0.9299	0.9501	0.9399
ConvMixer-NLA	0.9415	0.9460	0.9501	0.9481
ConvMixer-ECA	0.9452	0.9469	0.9640	0.9554

**Table 3:** Comparison of the proposed ConvMixer-ECA model with findings from the literature.

Author	Year	Dataset	Method	Accuracy (%)
Tenev et al. [38]	2014	50 healthy, 67 ADHD	SVM and voting	82.3
Khoshnoud et al. [39]	2015	10 healthy, 12 ADHD	LLE, ApEn, PNN	87.5
Mohammadi et al. [40]	2016	30 healthy, 31 ADHD	MLP neural network	93.65
Chen et al. [18]	2019	57 healthy, 50 ADHD	Deep CNN	90.29
Dubreuil-Vall et al. [15]	2020	20 healthy, 20 ADHD	Spectrogram and CNN	88.0
Tosun [16]	2021	16 subject	Data augmentation, PSD, SE, and LSTM	92.15
Saini et al. [17]	2022	80 healthy, 77 ADHD	PCA, KNN	86.0
Our proposed approach	2024	60 healthy, 61 ADHD	ConvMixer with ECA	94.52

## Data Availability

We thank the numerous contributors to the IEEE DataPort for their efforts in the collection, organization, and sharing of their datasets. The data that support the findings of this study are openly available in the IEEE DataPort at https://ieee-dataport.org/open-access/eeg-data-adhd-control-children (accessed on 9 April 2024).
